# Supplementary material for: Interfacial solute flux promotes emulsification at the water|oil interface
Source: Nat Commun. 2023 Feb 9;14:705. doi: 10.1038/s41467-023-35964-9 (PMC9911786; doi:10.1038/s41467-023-35964-9)
Supplement: Supplementary file 1 — Supplementary Information [file 41467_2023_35964_MOESM1_ESM.pdf]

Supplementary Information

Interfacial Solute Flux Promotes Emulsification at the Water|Oil Interface

Colón-Quintana, et al.

Table Of Contents:

1. Supplemental Table 1: Ion transfer potentials of relevant ions across the water|1,2-dichloroethane (DCE) interface…………………………………………………………………………….....S2
2. Supplemental Figure 1: Optical representation of partitioning of chloroauric acid over time… S3
3. Supplemental Figure 2: Example of the methods used for the determination of droplet size distribution and distance measurements………………………………………………………S4
4. Supplemental Figure 3: Histograms showing the frequency and average cross-sectional area of droplets as a function of distance from the interface…………………………………………S5
5. Supplemental Figure 4: Stability measurements of DCE droplets formed in water using dynamic light scattering…………………………………………………………………………..S6
6. Supplemental Figure 5: Images of bulk water and oil phases in contact showing the stability of the emulsion over time…………………………………………………………………….....S7
7. Supplemental Figure 6 and Supplementary Table 2: Reproducibility measurements of DCE droplets formed in water using dynamic light scattering………………………………………...S8
8. Supplemental Figure 7: Experimental controls for the effect of mutual saturation on the emulsification behavior……………………………………………………………………..S9
9. Supplemental Figure 8: Experimental controls for the effect of NaCl vs. KCl on emulsification behavior ………………………………………………………………………………….S10
10. Supplemental Figure 9: Experimental controls for the effect of NaCl on emulsification behavior.……………………………………………………………………………………….S11
11. Supplemental Figure 10: Determination of the effect of different ratios of $[Cl^-]_{(aq)}$:$[ClO_4^-]_{(aq)}$ on emulsion behavior……………………………………………………………………….....S12
12. Supplemental Figure 11: Flux-induced emulsification using other solvents …………………...S13
13. Supplemental Figures 12 and 13: Partition Coefficient Determination……………………….S14
14. Supplementary References ……………………………………………………………...S16

Supplementary Information

Interfacial Solute Flux Promotes Emulsification at the Water|Oil Interface

Colón-Quintana et al.

Ion:	$\Delta G^0_{w \to DCE}$ (kJ/mol)	$\Delta\varphi^0_{w \to DCE}$ (mV)
H^+	60.5 (1)	–627
Na^+	58.9 (1)	–610
K^+	52.9 (1)	–548
NEt_4^+	4.2 (2)	–44
NBu_4^+	–21.8 (2)	226
Cl^-	46.4 (2)	–481
ClO_4^-	17.2 (2)	–178
Br^-	38.5 (2)	–399
PF_6^-	8.7 (1)	–90

Supplementary Table 1: The standard Gibbs free energy of ion transfer from water (w) to DCE, as reported in Supplementary References (1) and (2), and the standard ion transfer potentials of these ions from water to DCE.

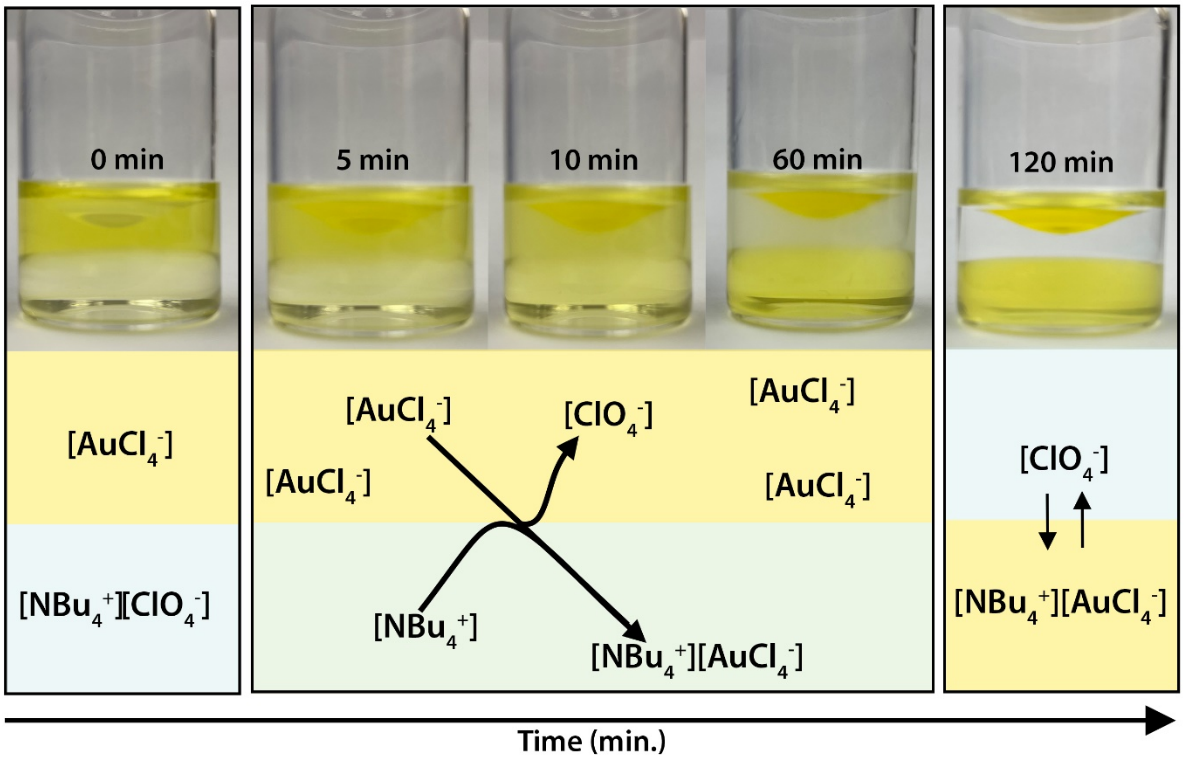

Supplementary Figure 1: Optical representation of partitioning of chloroauric acid over time. Initial concentrations consisted of an aqueous solution containing 10 mM $HAuCl_4$ and 1 M KCl (top layer) and a 1,2-dichloroethane/0.1 M $[NBu_4][ClO_4]$ (bottom layer). Images were taken at different timepoints to show progression of partitioning over time, with a schematic representation of the partitioning process below each corresponding image. Images were taken with a 12-megapixel camera with a f/2.0 aperture.

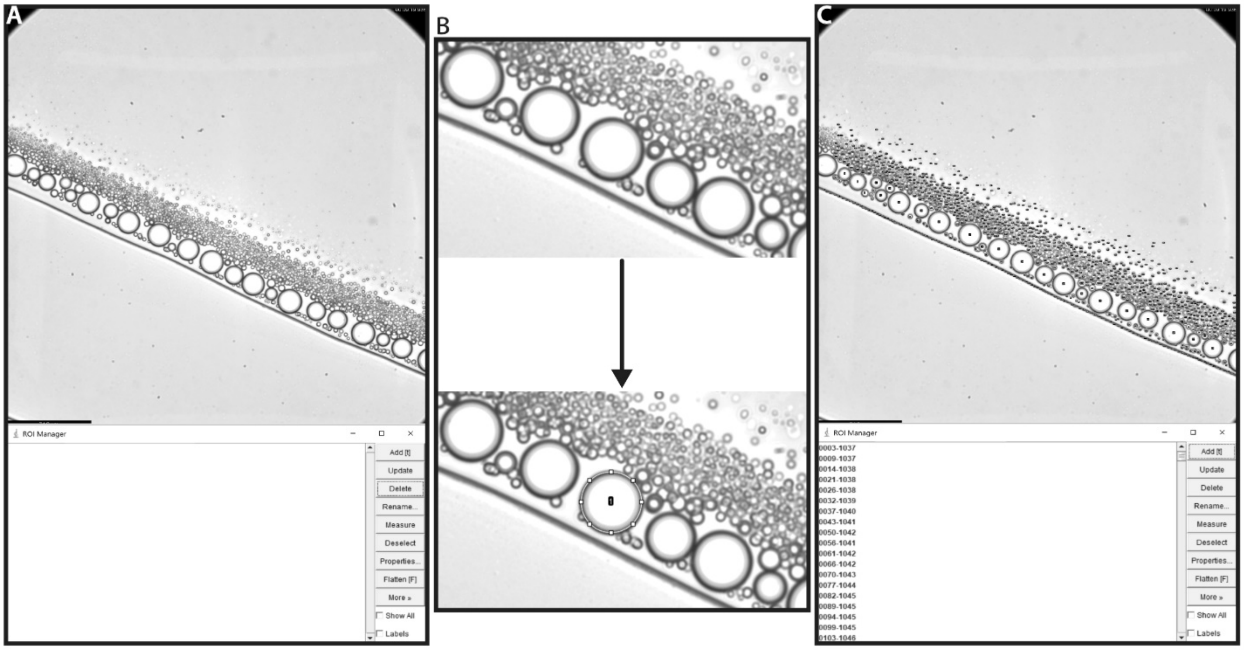

Supplementary Figure 2: Example of the methods used for the determination of droplet size distribution and distance measurements. A) Initial image prior to measurements with ImageJ software, the top image shows a representative droplet distribution after emulsification at the interface (10 mM $HAuCl_4$ in water, 0.1 M $[NBu_4][ClO_4]$ in DCE), with the bottom image showing ImageJ's region of interest (ROI) manager. B) Droplet measurement process using the ROI manager, where each droplet was individually selected, and hand measured to the closest ellipse. C) Image post measurements with ImageJ software. The top image shows all droplets identified and measured, while the bottom image shows completed ROI for measurements.

Droplet measurements were conducted using ImageJ's image analysis software. Images for the emulsion formation at the interface were opened and analyzed with the region of interest (ROI) manager tool. The scale bar and pixel vs. distance scale was set prior to measurements by calibrating the internal scale to the scale bar provided within each image by the microscope software. Each droplet was then individually added as a site of interest with its shape approximated to the nearest ellipse. The area and centroid position were then recorded for each individual drop after all ROI's were identified using the measure tool. Additionally, the centroid position of individual points (approximately 1 μm apart) along the liquid|liquid interface was measured. After measurement, all data was exported, and droplets were sorted according to their size and distance to the interface. The centroid position of each droplet was obtained within ImageJ and then the closest distance from the center of each droplet to the interface was calculated by use of the Pythagorean theorem, for all possible points. Finally, the shortest calculated hypotenuse was used as the reported distance to the interface.

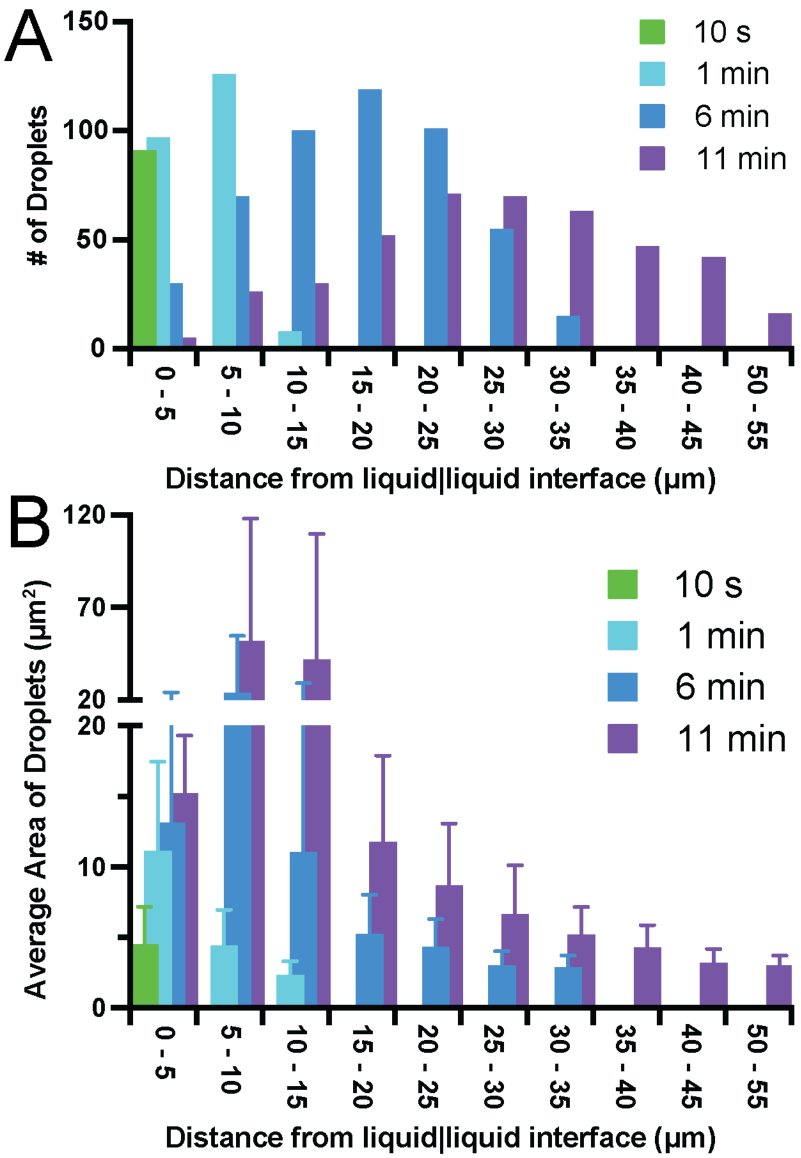

Supplementary Figure 3: Histograms showing the frequency (A) and average cross-sectional area (B) of droplets identified in images (Figure 1) as a function of distance from the interface at 10 s (green), 1 min (light blue), 6 min (dark blue), and 11 min (purple) after initial contact. All error bars in (C) are plotted as the standard deviations about the mean for N equal to the number of droplets identified in that region (from A).

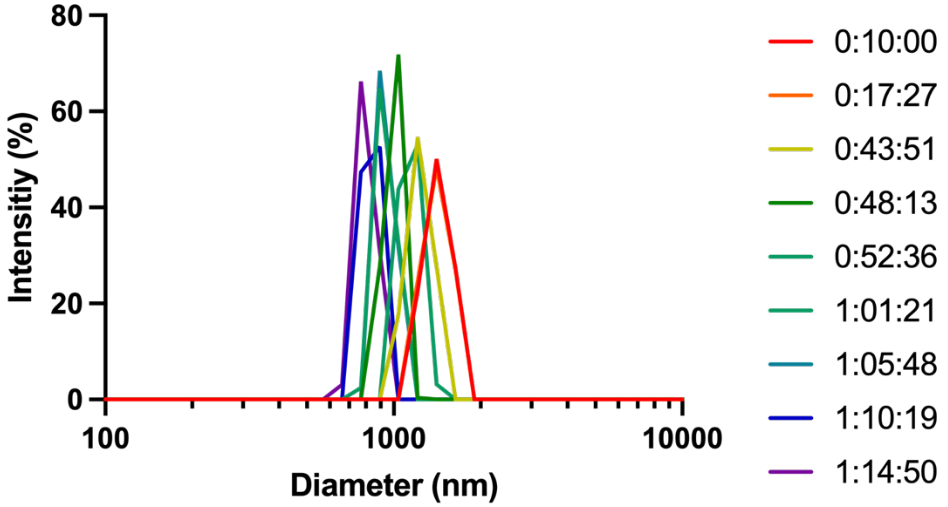

Supplementary Figure 4: Dynamic light scattering measurements of DCE droplets spontaneously formed in the aqueous phase when 10 mL $HAuCl_4$ was pipetted over 10 mL of DCE containing 0.1 M $[NBu_4][ClO_4]$. An overhead stirrer was inserted into the aqueous phase and was used to induce convection in the aqueous phase without making contact with the liquid|liquid boundary. Droplets were collected after ten minutes of solution contact and measured for over an hour. Monomodal distributions are observed for all cases with the droplet diameters decreasing with time. Source data are provided as a Source Data file.

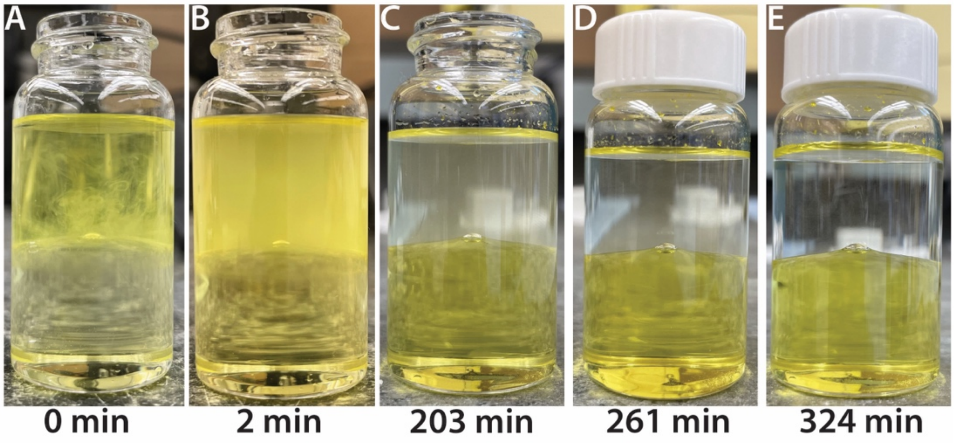

Supplementary Figure 5: Images for the stability of the observed emulsion over time. A 10 mM $HAuCl_4$ aqueous solution was pipetted over a 0.1 M $[NBu_4][ClO_4]$ DCE solution. The emulsion was allowed to form spontaneously at t = 0 min, afterwards overhead stirring was used for 1 min to induce convection and maximize emulsion formation. The observed emulsion was then monitored over time at A) 0 min, B) 2 min, C) 203 min, D) 261 min, and E) 324 min. A noticeable emulsion can be seen up to 261 min showing stability over long periods of time.

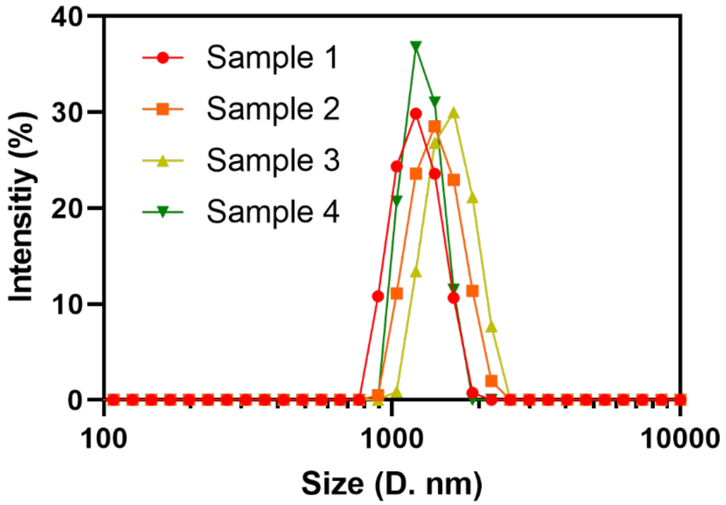

Supplementary Figure 6: Droplet size reproducibility measurements using dynamic light scattering. DCE droplets were spontaneously formed in the aqueous phase when 2 mL of 10 mM $HAuCl_4$ was pipetted above 2 mL of DCE containing 0.1 M $[NBu_4][ClO_4]$. All droplet dynamic light scattering measurements were taken 5 minutes after contact of both solutions for an N = 4. Source data are provided as a Source Data file.

	Sample 1	Sample 2	Sample 3	Sample 4	Combined Avg. Size
Average Size (nm)	1230.5	1441.0	1611.8	1283.9	1391.8
Std. Dev. (nm)	135.30	157.11	182.95	187.08	171.72
RSD (%)	10.995	10.902	11.350	14.571	12.337

Supplementary Table 2: Data and statistical analysis for droplet size and reproducibility measurements using dynamic light scattering (Supplementary Figure 6). The average size, standard deviation, and relative standard deviation are provided for all samples measured and for a combined average size.

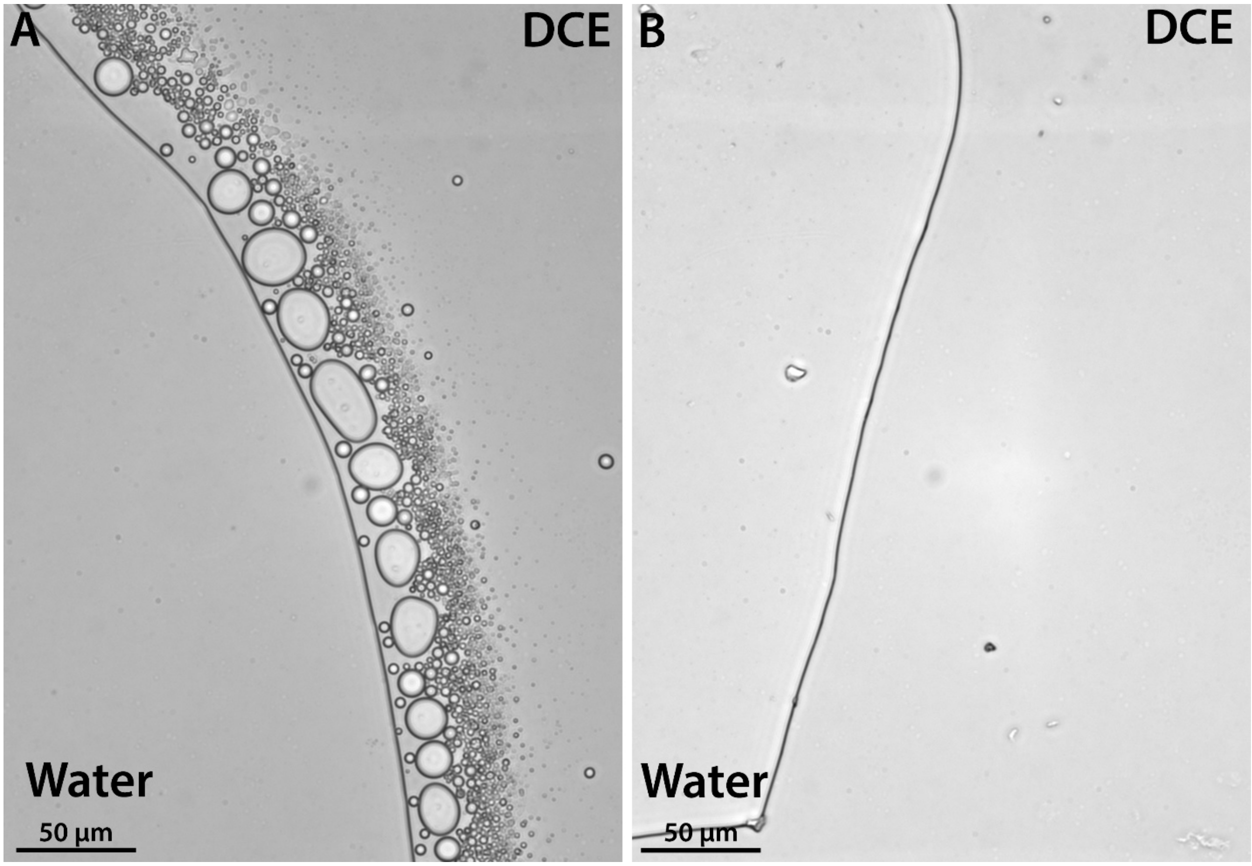

Supplementary Figure 7: Experimental controls for the effect of mutual saturation on the emulsification behavior. To test the effect of saturation on our system, mutually saturated solutions of water and DCE were tested for each phase. A water-saturated DCE phase was used as the organic phase and a DCE-saturated water solution was used for aqueous phase for both A and B. Image A contained 10 mM $HAuCl_4$ with 1 M NaCl aqueous solution and 0.1 M $[NBu_4][ClO_4]$ DCE solution, while Image B contained only NaCl and $[NBu_4][ClO_4]$ (no $HAuCl_4$). The images shown were taken after 10 minutes of solutions being in contact. All optical micrographs were taken with a 40x NA 0.60 objective and a 500 ms exposure time.

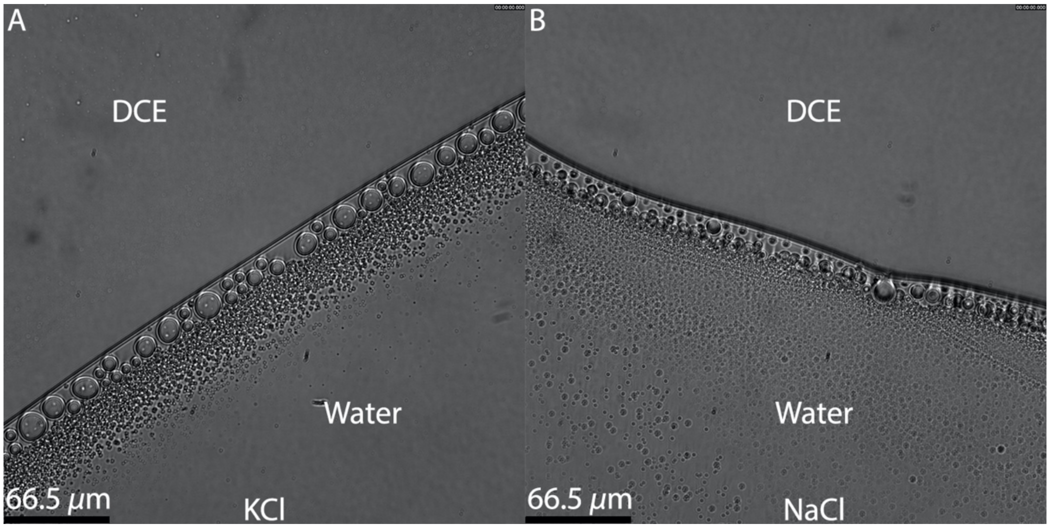

Supplementary Figure 8: Experimental control for the effect of NaCl vs. KCl on emulsification behavior. The DCE phase contained 0.1 M $[NBu_4][ClO_4]$ and the aqueous phase contained 10 mM $HAuCl_4$ and 1 M KCl (A) or 1 M NaCl (B). A microemulsion still formed at the liquid|liquid boundary, indicating that using NaCl vs. KCl in the aqueous phase has little effect on the spontaneous emulsification. These images were taken with a 40x NA 0.60 objective and a 500 ms exposure time.

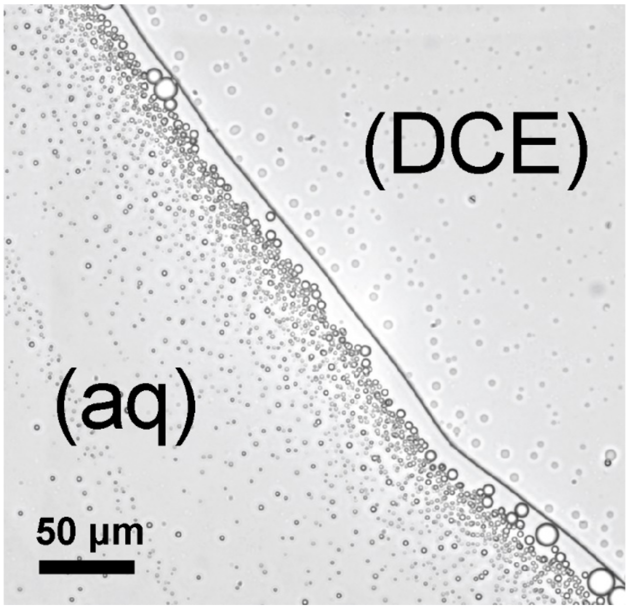

Supplementary Figure 9: Experimental control for the effect of NaCl on emulsification behavior. The DCE phase contained 0.1 M $[NBu_4][ClO_4]$ and the aqueous phase contained 10 mM $HAuCl_4$ for this experiment. No additional supporting electrolyte was present in the aqueous phase (i.e., no sodium chloride). A microemulsion still formed at the liquid|liquid boundary, indicating that neither Na^+ nor Cl^- are necessary for the spontaneous emulsification. This image was taken 10 minutes after the solutions were in contact. These images were taken with a 40x NA 0.60 objective and a 500 ms exposure time.

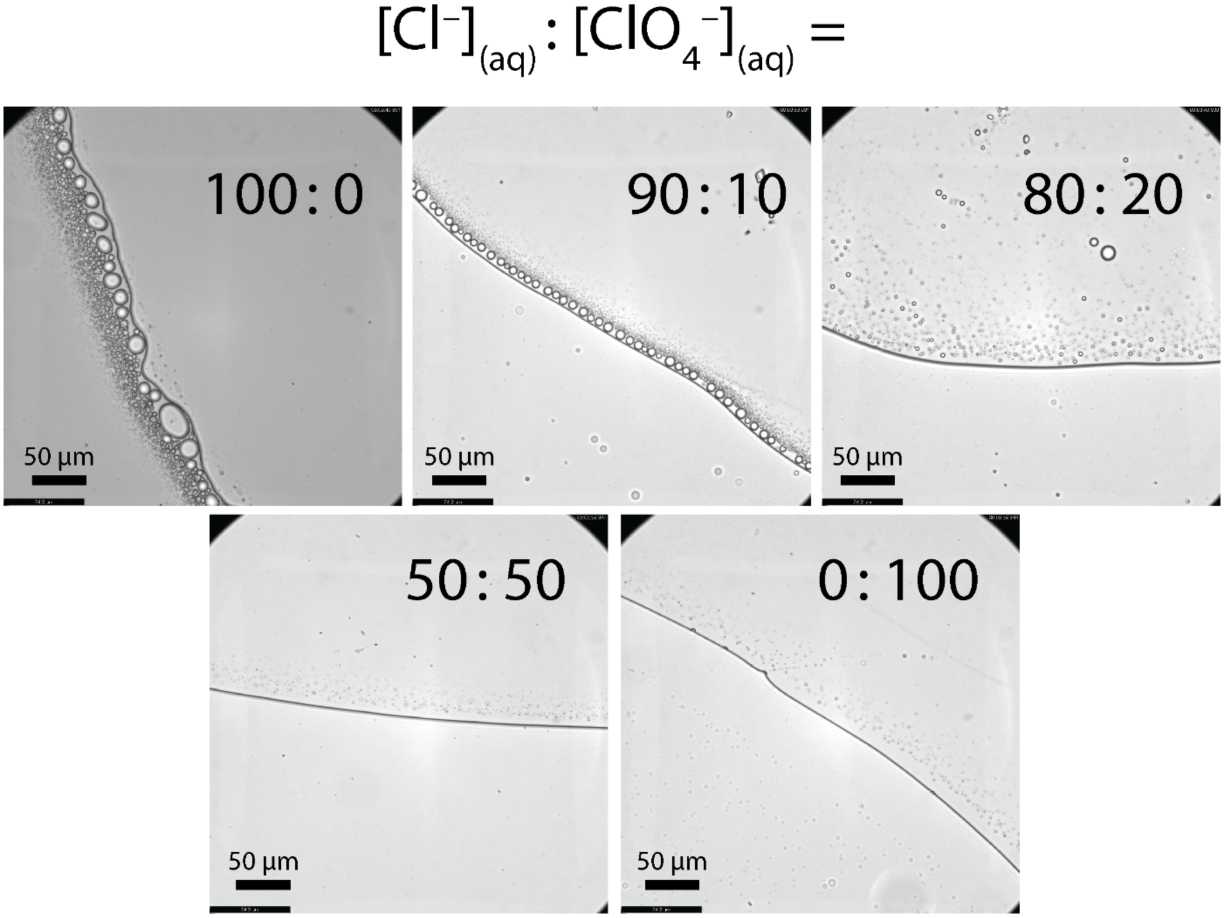

Supplementary Figure 10: Determination of the effect of different ratios of $[Cl^-]_{(aq)}$:$[ClO_4^-]_{(aq)}$ on emulsion behavior. The DCE phase contained 0.1 M $[NBu_4][ClO_4]$ for all experiments. The aqueous phase contained 10 mM $HAuCl_4$ with different ratios of sodium chloride and sodium perchlorate (but always 1 M total supporting electrolyte concentration). The images are taken 10 minutes after solutions were in contact.

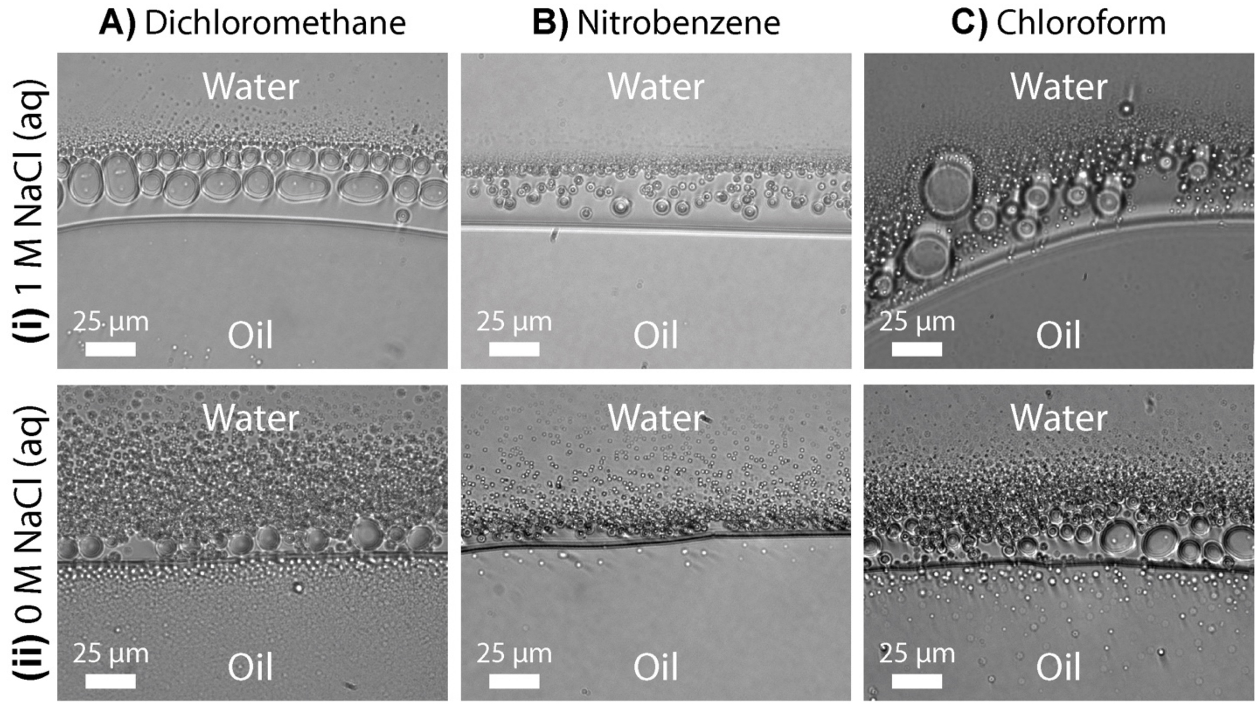

Supplementary Figure 11: Light microscopy images taken at the water|DCE boundary when the aqueous phase contained 10 mM $HAuCl_4$ and the oil phase contained 0.1 M $[NBu_4][ClO_4]$ for (A) dichloromethane, (B) nitrobenzene, and (C) chloroform with either 1 M NaCl (i) or 0 M NaCl (ii) present in the aqueous phase. The water and DCE phases are indicated, and the scale bars are 25 µm. All optical micrographs were taken with a 40x NA 0.60 objective and a 500 ms exposure time.

All partitioning coefficients were measured experimentally by use of UV-VIS for thiocyanate and by use of electrochemical methods for ferricyanide. Here an equal volume of either 10 mM potassium thiocyanate or 10 mM potassium ferricyanide was added to a vial containing an equal volume of 0.1 M [NBu_4][ClO_4] solution. These two phases were then vigorously mixed for 5 min to maximize mixing and partitioning between the two phases. Each vial was then let still for about 2 hr to allow the solutions to reach equilibrium and allow phase separation due to differences in density. This was repeated for an N = 4.

Thiocyanate concentrations and partitioning coefficients were determined by use of UV-VIS spectrometry. Both known and unknown concentrations of thiocyanate were then complexed known concentration of $Fe(NO_3)_3$. The colored complex was then measured with UV-VIS spectroscopy to identify the degree of partitioning by use of a calibration curve. The difference in concentration prior and post partitioning was then used to identify its partitioning coefficient.

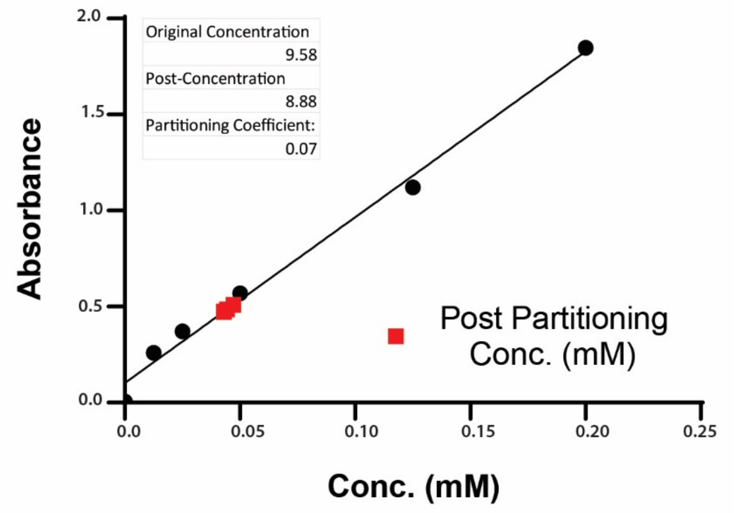

Supplementary Figure 12: Graph for the determination of the partitioning coefficient of thiocyanate in water when in the presence of tetrabutylammonium perchlorate in DCE using UV-VIS Spectroscopy. A 10 mM KSCN aqueous solution was allowed to partition for 2 hr in the presence of an equal volume of 0.1 M tetrabutylammonium perchlorate in DCE. Resulting thiocyanate solutions were then exposed to an excess of $Fe(NO_3)_3$ to form a colored complex that was then measured with UV-VIS spectroscopy to identify the degree of partitioning. Solutions were diluted accordingly to meet limitations of UV-VIS, and the concentrations were corrected by their dilution factor. The original concentration was measured to be 9.58 mM and the post-partitioning concentration was measured to be 8.88 mM. Good linearity for all concentrations was observed. No noticeable variation was observed for the points within the curve shown, all points were measured for a minimum N = 3.

For the identification of the partitioning coefficient of ferricyanide, the concentration of ferricyanide in the aqueous phase was measured prior and post partitioning. This was measured by use of cyclic voltammetry with a r = 6.25 µm microelectrode. Supplementary Figure 13 shown below demonstrates representative voltammetry. Source data are provided as a Source Data file.

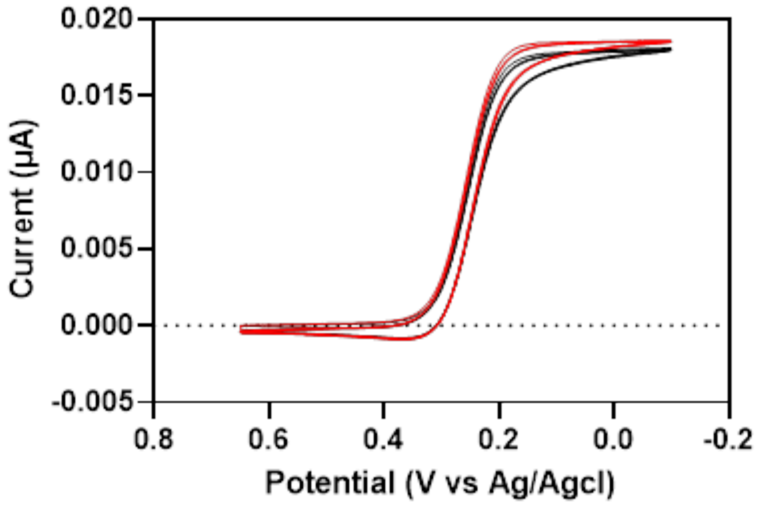

Supplementary Figure 13: Cyclic voltammetry for 10 mM potassium ferricyanide in 1 M KCl and 0.1 M $[NBu_4][ClO_4]$ DCE for partition coefficient measurements. The red trace corresponds to the concentration of analyte of interest before mixing. The CV was taken from -0.1 V to 0.7 V vs. Ag|AgCl prior to mixing. The black trace corresponds to measurements in the aqueous phase after mixing. Measurements were made with a r = 6.25 µm gold microelectrode, a r = 0.5 mm glassy carbon rod counter and a Ag|AgCl reference electrode at a 50 mV/s scan rate. The concentration was obtained by use of the difference in limiting current of both traces, where the partition coefficient was obtained to be Kp = 0.03. Source data are provided as a Source Data file.

Supplementary References

(1) Zhou, M.; Gan, S.; Zhong, L.; Su, B.; Niu, L. Ion Transfer Voltammetry by a Simple Two Polarized Interfaces Setup. *Analytical Chemistry* **2010**, *82* (18), 7857-7860. DOI: 10.1021/ac102010b.

(2) Markin, V. S.; Volkov, A. G. The gibbs free energy of ion transfer between two immiscible liquids. *Electrochimica Acta* **1989**, *34* (2), 93-107. DOI: https://doi.org/10.1016/0013-4686(89)87072-0.
